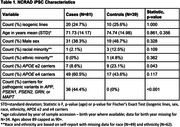# NCRAD iPSCs: a vital resource for the Alzheimer’s disease research community

**DOI:** 10.1002/alz.092789

**Published:** 2025-01-03

**Authors:** Kelly N. Nudelman, Trever Jackson, Jeanine D. Marshall, Kelley M. Faber, Sarah K Ohlemacher, Michael C. Edler, Tatiana M. Foroud, Jason S. Meyer

**Affiliations:** ^1^ National Centralized Repository for Alzheimer’s Disease and Related Dementias (NCRAD), Indianapolis, IN USA; ^2^ Department of Medical and Molecular Genetics, Indiana University School of Medicine, Indianapolis, IN USA; ^3^ Indiana Alzheimer’s Disease Research Center, Indianapolis, IN USA

## Abstract

**Background:**

The National Centralized Repository for Alzheimer’s Disease and Related Dementias (NCRAD) is continuing to develop a bank of induced pluripotent stem cells (iPSCs) that are available by request to the Alzheimer’s disease (AD) research community.

**Methods:**

As part of the pipeline for quality control of received cell lines, DNA was extracted for all lines and was submitted for whole genome sequencing (WGS). Paired‐end WGS data was generated using the Illumina NovaSeq 6000 and processed following GATK best practices using the Sentieon pipeline. WGS data was annotated with Annovar, and data was reviewed for reported cell line variants and checked with Varsome and Franklin. Sequencing data was reviewed for all nonsynonymous and splicing variants in the *APP*, *PSEN1*, *PSEN2*, *GRN*, and *MAPT* genes. Additionally, DNA from cell lines was genotyped in‐house by NCRAD to generate apolipoprotein E (APOE) genotypes, and this data was compared with the WGS to confirm sample identity. Basic clinical and demographic data was also collected, including sex, case/control status, age, race, and ethnicity.

**Results:**

To date, DNA has been extracted and genotyped at NCRAD for lines from 183 participants including generation of *APOE* genotypes passing quality control. Of these, 120 cell lines have returned WGS data passing quality control. **Table 1** describes the demographic and clinical features for these lines, which include data for lines from 90 individuals as well as data for 30 isogenic lines. Of the 120 lines with available WGS, there are 13 case *APP* variant carriers, 13 case *MAPT* variant carriers, 8 case *PSEN1* variant carriers, and 2 case *PSEN2* variant carriers. Additionally, these cell lines included two control carriers of variants of uncertain significance (VUS) in *GRN* or *PSEN2*, as well as two cases carrying VUS in *PSEN1* or *APP*.

**Conclusions:**

NCRAD continues to expand iPSCs for the research community; adding WGS data to this resource provides an expanded scope for pre‐screening as well as functional research. Future directions include review of variants being tested in the Model Organism Development & Evaluation for Late‐Onset AD (MODEL‐AD) to provide additional value to researchers.